# Physicochemical Study of the Self-Disintegration of Calcium Orthosilicate (β→γ) in the Presence of the C_12_A_7_ Aluminate Phase

**DOI:** 10.3390/ma14216459

**Published:** 2021-10-28

**Authors:** Michał Pyzalski, Jarosław Dąbek, Anna Adamczyk, Tomasz Brylewski

**Affiliations:** Faculty of Materials Science and Ceramics, AGH University of Science and Technology, A. Mickiewicza 30 Street, 30-059 Kraków, Poland; dabek@agh.edu.pl (J.D.); aadamcz@agh.edu.pl (A.A.); brylew@agh.edu.pl (T.B.)

**Keywords:** calcium orthosilicate, self-disintegration, polymorphic transformation

## Abstract

The β-γ polymorphic transition of calcium orthosilicate (C_2_S) is a key phenomenon in cement chemistry. During this transition, the compound expands due to structural changes and a significant reduction in its density is observed, leading to its disintegration into a powder with a very high specific surface area. Owing to this tendency of the C_2_S material to “self-disintegrate”, its production is energy-efficient and thus environmentally friendly. A physicochemical study of the self-disintegration process was conducted with the aim of determining how the amount of dodecacalcium hepta-aluminate (C_12_A_7_) in calcium orthosilicate (C_2_S) affects the temperature at which the polymorphic transi-tions from α’L-C_2_S to β-C_2_S and from β-C_2_S to γ-C_2_S undergo stabilization. The applied techniques included differential thermal analysis (DTA), calorimetry and X-ray diffraction (XRD), and they made it possible to determine what C_2_S/C_12_A_7_ phase ratio in the samples and what cooling rate constitute the optimal conditions of the self-disintegration process. The optimal cooling rate for C_2_S materials with a C_12_A_7_ content of up to 60 wt% was determined to be 5 K·min^−1^. The optimal mass ratio of C_2_S/C_12_A_7_ was found to be 70/30, which ensures both efficient self-disintegration and desirable grain size distribution.

## 1. Introduction

Calcium orthosilicate with the formula Ca_2_[SiO_4_] (2CaO·SiO_2_) and—more specifically—as a solid solution known as belite and referred to as C_2_S, is one of the four main constituent phases of the currently manufactured Portland cements. It is also found in alkali-activated blast furnace slag [1], steel slag [2], or hydraulic lime [3], in which it also serves the role of the main component that gives the material its hydraulic properties. The ubiquitous presence of calcium orthosilicate in binding material technology stems from its desirable physicochemical properties, which are themselves a consequence of the differences in the structure of its polymorphs.

**Figure 1:**
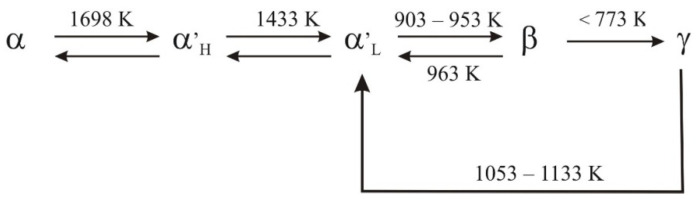
Polymorphic transitions of Ca_2_[SiO_4_] [4].

Ca_2_[SiO_4_] forms five phases. The first four phases are designated α, α’_H_, α’_L_ and γ, and they are stable at different temperatures and under ambient pressure. The fifth phase, namely β, is the most important with regard to hydraulic properties, and it is thermodynamically stable only under high pressure. Figure 1 shows the generally accepted scheme of polymorphic transitions of calcium orthosilicate, which was proposed by Taylor [4].

The common feature of all polymorphs of this oxide is the presence of free oxysilicate tetrahedrons—[SiO_4_]^4−^—joined together by calcium atoms located at octahedral sites, whereas their structural differences stem from the different relative positions of the above-mentioned tetrahedrons and octahedrons.

**Figure 2:**
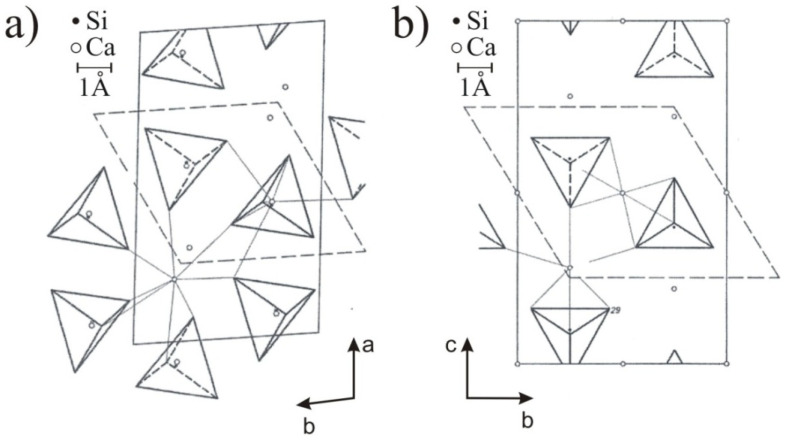
Crystal structures of calcium orthosilicate polymorphs: (**a**) β-C_2_S, (**b**) γ-C_2_S [7].

As far as cement chemistry is concerned, the most significant transition is the monotropic transition of the β-C_2_S polymorph into γ-C_2_S, often referred to as the self-disintegration of calcium orthosilicate. The β-C_2_S crystallizes in a monoclinic system, and its unit cell—as in the case of the unit cells of α‘_L_-Ca_2_S and α‘_H_-C_2_S—contains four Ca_2_[SiO_4_] molecules. The oxysilicate tetrahedrons in β-Ca_2_S are not located in any element of symmetry, and they are rotated by 30° relative to the [SiO_4_]^4−^ tetrahedrons in α‘_L_-C_2_S [5]. They are bound to calcium cations with different coordination. Half of the Ca^2+^ ions have a coordination number (C.N.) of 6, and they are alternately placed below and above the [SiO_4_]^4−^ tetrahedrons, while the remaining calcium cations are found in the voids between the oxysilicate tetrahedrons and have a C.N. of 8 [5]. The γ-C_2_S polymorph, on the other hand, has a structure with a orthorhombic symmetry, which is similar to that of olivine. The unit cell of this phase contains four Ca_2_[SiO_4_] molecules. The oxysilicate tetrahedrons are located alternately on the plane of the *ab* plane. All calcium ions in this phase have an octahedral coordination. Half of all Ca-centered octahedrons are located in inversion centers, whereas the other half are found on the *ab* plane, at a small distance from the glide plane [6]. As can be seen from the comparison of the crystal structures of the polymorphic phases shown in Figure 2, the transition from β-C_2_S to γ-C_2_S requires a change in the structure, namely a change in the spatial orientation of the [SiO_4_]^4−^ tetrahedrons and the displacement of calcium cations, which ultimately lead to expansion in the direction of the *c* axis [7]. The transition results in a significant drop in the density of calcium orthosilicate and thereby its disintegration.

The self-disintegration phenomenon is well-known and is of fundamental importance in Grzymek’s method of producing aluminum oxide and Portland cement with high alite content. This complex method based on sintering and self-disintegration has been the subject of numerous studies and patents [8,9,10,11,12,13,14]. The method allows aluminate to be obtained from waste materials that contain it, including fly ashes, coal found in shales, and post-extraction waste formed during the production of aluminate by means of the Bayer method [15,16]. In Grzymek’s method, the appropriate proportions of the above-mentioned waste materials undergo thermal treatment during which the obtained sinters self-disintegrate. This transition is associate with a 10 percent increase in the specific density of calcium orthosilicate and causes the sinter to autodisperse. The powder formed as a result exhibits a very highly developed surface area, which is ca. 10,000 cm^2^·g^−1^ (Blaine).

**Figure 3:**
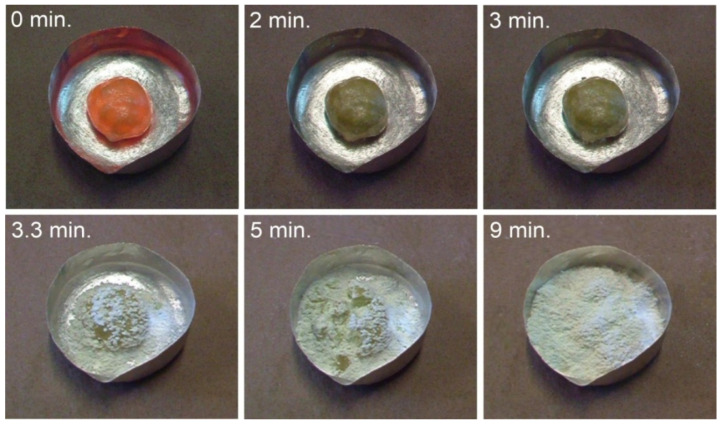
Polymorphic transition from β-C_2_S to γ-C_2_S, illustrating the complete self-disintegration over time.

Figure 3 shows photographs that present the subsequent stages of the self-disintegration of calcium orthosilicate during the transition from β-C_2_S to γ-C_2_S. The Supplementary Materials section includes a short movie that shows the complete self-disintegration of the material.

The extraction of aluminum in an aqueous solution yields an intermediate product for the production of aluminum and some residual material, which is utilized in another stage during which Portland cement with high alite content is obtained [16]. The thermal treatment of a set of raw materials that includes different amounts of aluminate phases such as mayenite, which has a composition of 12CaO·7Al_2_O_3_ and is denoted as C_12_A_7_, favor the formation of a considerable amount of a liquid phase consisting mostly of melted calcium aluminates. The occurrence of this liquid phase significantly affects the rate of C_2_S synthesis and the dynamics of the polymorphic transition from β-C_2_S to γ-C_2_S during the cooling process and, subsequently, the rate of the self-disintegration of the sinter [17,18,19].

It is currently thought that the direction and rate of the polymorphic transition from β-C_2_S to γ-C_2_S are determined by the formation rate of crystallization nuclei of new phases as well as their growth rate [20]. While the formation process itself is slow and depends on the reaction medium, the growth of these nuclei is rapid [21].

The research conducted so far indicates that the course of the transition from β-C_2_S to γ-C_2_S is significantly affected by the size of the crystals of the β-C_2_S phase [22]. Studies have shown that when the size of the crystals of the β-C_2_S phase does not exceed 5 μm, the formation of the γ-C_2_S phase is prevented. In such conditions, a metastable β-C_2_S polymorph resistant to self-disintegration forms [22].

On the other hand, crystals with a size of 30 μm contain γ-C_2_S inclusions, which promote the self-disintegration of C_2_S. Reports have demonstrated that both the temperature and duration of thermal treatment significantly affect the transition from β-C_2_S to γ-C_2_S [23]. The transition does not occur if the temperature of synthesis is below 1698 K. This phenomenon is usually explained by “vacant” spaces that limit the growth of fine crystals of the β-C_2_S phase and subsequently the formation of γ-C_2_S nuclei in β-C_2_S crystals [22,23,24,25,26].

The appropriate selection of the synthesis conditions or the introduction of a stabilizing addition can yield polymorphic forms of C_2_S that are unstable under ambient temperature and pressure conditions [27]. The crystallochemical stabilization of polymorphs of C_2_S can be achieved by doping C_2_S with the stoichiometric composition with the following elements: As^2+^, P^5+^, B^3+^, Ba^2+^, Sr^2+^, Cr^6+^, V^5+^, Na^+^, K^+^ and Fe^3+^ [25,27].

The authors of papers [21,22,26] have shown that the β-C_2_S phase can be stabilized by substituting some of the [SiO_4_]^4−^ tetrahedrons with smaller anion complexes or by substituting some of the Ca^2+^ ions with cations with a larger ionic radius. Stabilization can also entail the formation of solid solutions; the role of anions such as [BO_4_]^5−^ and [PO_4_]^5−^ is particularly important in this respect. The stabilization of the β-C_2_S polymorph in paper [15] revealed correlations between the stabilizing properties of ions and their polarization capacity. The authors of this paper elaborated an empirical formula that expresses the dependence between the properties of the applied cations and the temperature at which C_2_S self-disintegration starts to occur.

The results presented in [23,26,27] focused on the stabilization of the polymorphic C_2_S transition responsible for self-disintegration after changing the pressure to 2000 atm. The lower end of the range in which the phase transition from α’_L_-C_2_S to β-C_2_S is possible was estimated to be 140 kg·cm^−2^, which corresponds to 948 K.

It was established that the change in the C_2_S/C_12_A_7_ phase ratio in a self-disintegrating sinter has a considerable influence on the mean temperature of the transition from β-C_2_S to γ-C_2_S and on the rate at which this process occurs [14].

Cooling a sinter composed of a mixture of C_2_S and C_12_A_7_ to a temperature below 773 K leads to the recrystallization of the glassy phase of calcium aluminate and the decomposition of the glassy layer of C_2_S. This results in a decrease in pressure and the elimination of internal stress in high-temperature C_2_S polymorphs. The elimination of internal stress leads to a rapid restoration of the phase equilibrium, which corresponds to the presence of the lower-temperature β-C_2_S polymorph, which undergoes spontaneous disintegration. The authors of papers [15] and [17] also came to the conclusion that the glassy phase layers that develop around C_2_S grains stabilize the metastable form of the β-C_2_S phase.

Based on the results presented in [16] it can be stated that cooling the sinters at a slow rate promotes the transition from β-C_2_S to γ-C_2_S. Increasing the cooling rate for sinters containing aluminate phases reduces the temperature at which the polymorphic transition from β-C_2_S to γ-C_2_S occurs, thereby affecting the self-disintegration of C_2_S. It should be emphasized that the presence of aluminate phases in the sinter in addition to the C_2_S phase favors the stabilization of the β-C_2_S polymorph during rapid cooling [18].

The above-cited studies indicate that the C_12_A_7_ aluminate phase plays a significant part in the polymorphic transition from β-C_2_S to γ-C_2_S. Moreover, of the five basic calcium aluminates in the CaO-Al_2_O_3_ phase system, namely C_3_A, C_12_A_7_, CA, CA_2_ and CA_6_, the C_12_A_7_ mayenite melts at the lowest temperature, i.e., ca. 1673 K [28], which is why it was determined to be the best choice for a study on the self-disintegration of calcium orthosilicate (β-γ).

In order to broaden the knowledge concerning the self-disintegration of C_2_S, the comprehensive explanation of the influence of the amount of liquid phase originating from C_12_A_7_ and thereby the determination of the optimal C_2_S/C_12_A_7_ phase ratio and cooling rate were included as objectives in the present study.

## 2. Materials and Methods

Sets of materials, each with a weight of 1 kg, were prepared using the following reagents: CaCO_3_, SiO_2_, and Al_2_O_3_, all of analytical grade purity and supplied by POCH Poland S.A (Gliwice, Poland). The composition of the prepared sets is shown in Table 1.

All prepared sets were homogenized for 24 h in a Deval drum (Cracow, Poland) to ensure sufficient homogeneity. The samples were thermally treated for 1 h in air at 1723 K in a sylite furnace (Cracow, Poland).

The chemical composition of the obtained sinters was analyzed according to the PN-EN 196-2 standard [29]. The results are presented in Table 2, and they show that the samples retained their nominal composition after sintering.

The obtained samples were examined by means of differential thermal analysis (DTA), which was performed using the LABSYS TGA/STA thermoanalyzer (Seteram, Lyon, France). Based on these analyses, the temperatures at which particular thermal effects occurred during the heating and cooling of samples were determined. The investigations were performed in argon and for samples weighing 0.5 g. The heating rate ranged from 5 to 10 K·min^−1^, and the cooling rate from 2.5 to 20 K·min^−1^. The exact values for particular investigations are given in the corresponding sections.

High-temperature calorimetry was used to determine the amount of heat absorbed or evolved during physicochemical processes that occurred during the heating and cooling of the examined samples. These investigations were performed for samples weighing 0.7 g. Thermal treatment was carried out in an argon atmosphere, with a heating rate of 5 K·min^−1^.

The applied research methods were supplemented with X-ray diffraction (XRD), which was used to determine the qualitative and quantitative phase composition of the samples. For this purpose, a Philips X-ray diffractometer (Philips, Eindhoven, Netherlands) equipped with a copper lamp, a PW 1140 high-voltage generator (Philips, Eindhoven, Netherlands), and a PW 1050/70 vertical goniometer (Philips, Eindhoven, Netherlands) was used. XRD measurements were carried out for all samples in the 10–60° 2Θ range, with a constant shift in the position of the goniometer arm equal to 0.05° 2Θ and a measurement time—3 s.

## 3. Results

### 3.1. Phase Composition of the Samples

**Figure 4:**
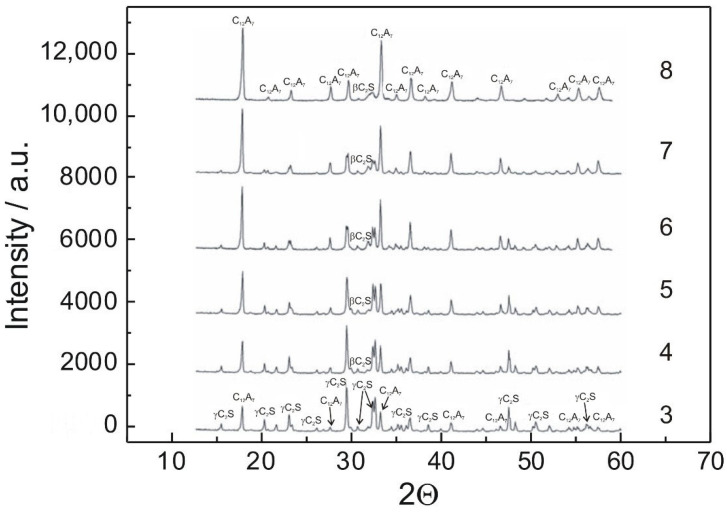
X-ray diffraction patterns obtained for samples 3, 4, 5, 6, 7 and 8.

Figure 4 shows the results of XRD analyses conducted for certain samples after 1 h of thermal treatment in air at 1723 K. These phase analyses confirmed the results of chemical analyses (Table 2). C_2_S and C_12_A_7_ were the only phases detected in the investigated samples.

### 3.2. Determination of the Melting Point of C_12_A_7_

**Figure 5:**
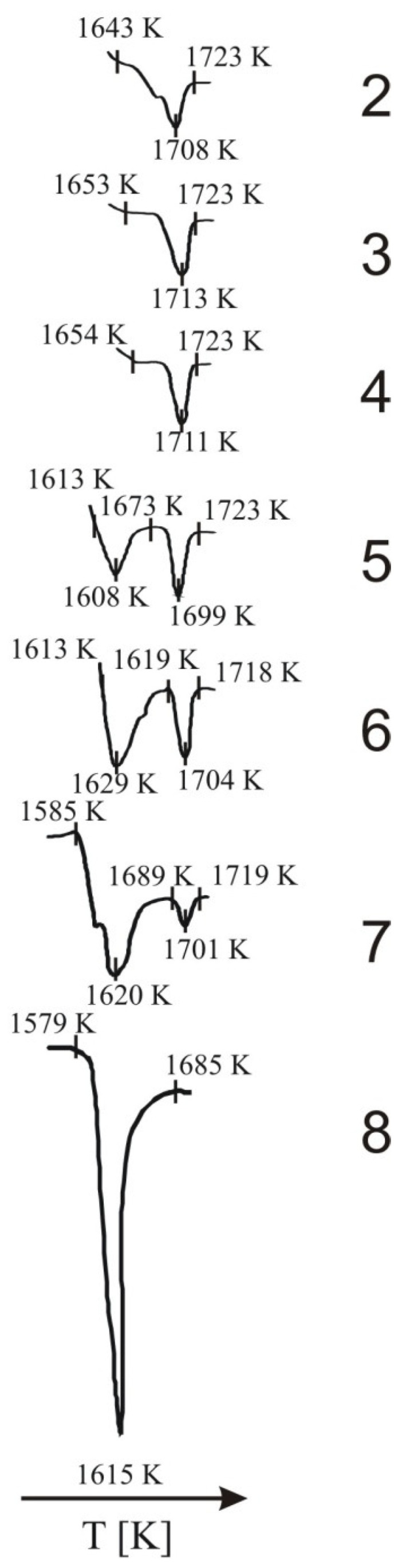
Thermograms showing the point at which the C_12_A_7_ in the tested samples melted.

Figure 5 shows thermograms representing the melting of the C_12_A_7_ phase in samples 2, 3, 4, 5, 6, 7 and 8, recorded while the samples were heated at a rate of 10 K·min^−1^.

The obtained data reveal that a pronounced endothermic effect occurred in the temperature range of 1613–1689 K; this effect was associated with the melting of the aluminate phase in samples 5–8, which contained a significant mass fraction of this phase. This effect was not observed for samples 2–4. In the range of 1673–1719 K, another endothermic effect occurred in all samples with the exception of sample 8. This effect can be presumed to be associated with the sintering process.

### 3.3. Determination of the Time of C_2_S Synthesis in the Presence of Different Amounts of C_12_A_7_

In order to obtain representative results for samples with various C_2_S/C_12_A_7_ ratios, investigations aimed at establishing the time required for the full synthesis of C_2_S at 1723 K were conducted. Selected samples were placed in a furnace and the above-specified temperature was set. The samples were heated for 5, 15, 30, 45 or 60 min, removed from the furnace, and left to cool in ambient conditions. The obtained self-disintegrated particulates were examined to determine the amount of free calcium oxide by means of the Franke method [30]. The results of these examinations for samples 1, 2, 4, 6, 7 and 8 are presented in Table 3.

In the case of sample 2, which had a C_12_A_7_ content of only 10 wt%, the amount of free calcium oxide decreased steadily with synthesis time. For samples 4, 6, 7 and 8, this amount was determined mostly by the amount of the liquid aluminate phase, since CaO content remained at a comparable, low level over the time interval from 15 to 60 min. From a practical standpoint, this means that even after as little as 15 min of synthesis it is possible to obtain a fully reacted material provided that the mass fraction of the C_12_A_7_ phase is at least 30 wt%.

### 3.4. Study of the Influence of C_12_A_7_ on the Transition fromβ-C_2_S to γ-C_2_S

The influence of the presence of the C_12_A_7_ phase on the course of the polymorphic transition from C_2_S was determined using differential thermal analysis. The first stage involved the controlled heating of the measurement setup and the sample to 1723 K. The samples were exposed to this temperature for 60 min and then cooled at a rate of 2.5 K·min^−1^. During the cooling process, thermograms were recorded and the amount of evolved heat was determined using the calorimetric method.

**Figure 6:**
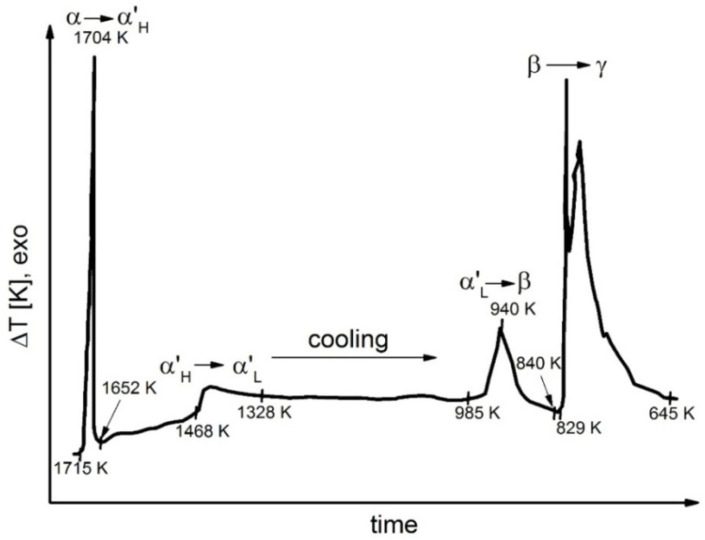
Thermogram recorded for sample 1 during the cooling process.

**Figure 7:**
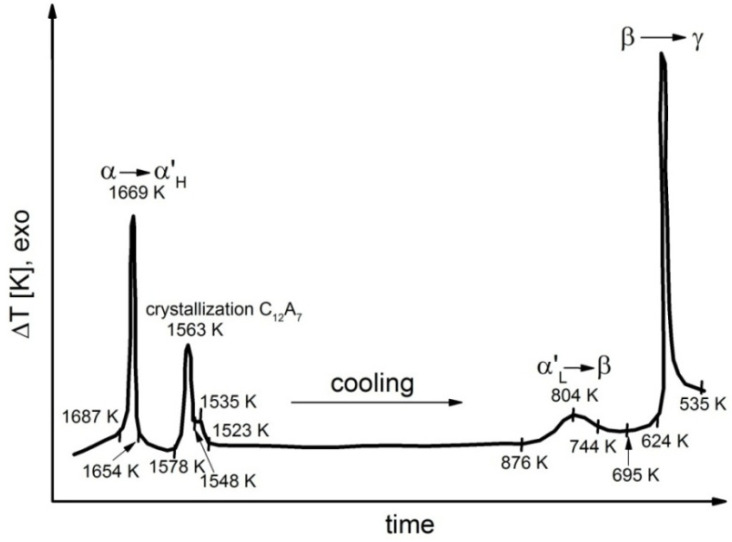
Thermogram recorded for sample 4 during the cooling process.

**Figure 8:**
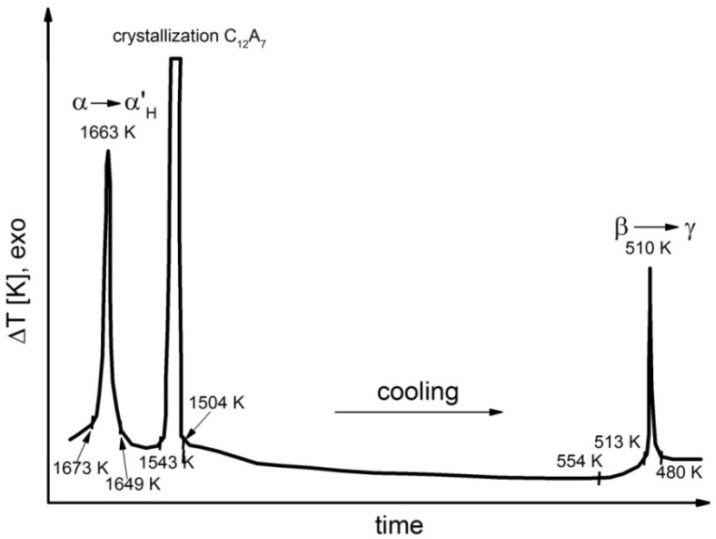
Thermogram recorded for sample 6 during the cooling process.

**Figure 9:**
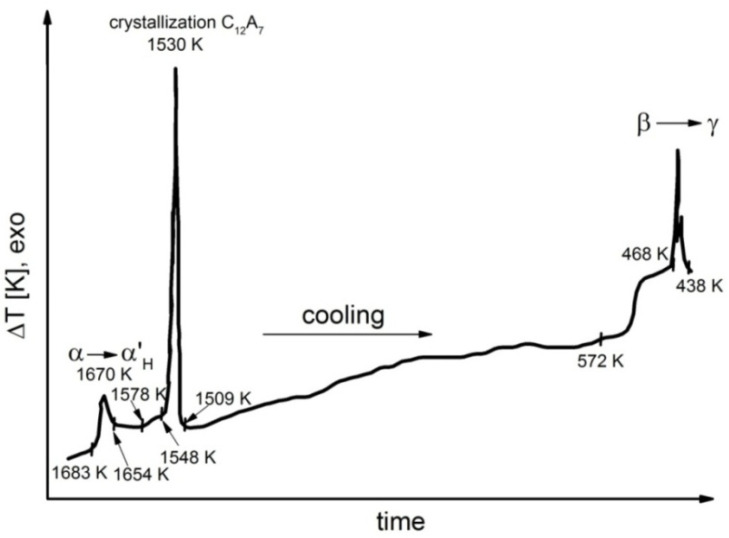
Thermogram recorded for sample 7 during the cooling process.

**Figure 10:**
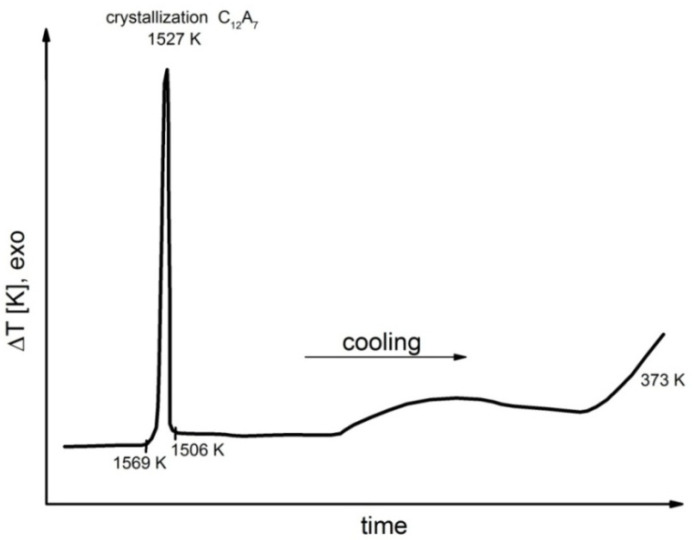
Thermogram recorded for sample 8 during the cooling process.

Representative results of DTA performed during the cooling process for samples 1, 4, 6, 7 and 8 are shown in Figure 6, Figure 7, Figure 8, Figure 9 and Figure 10.

**Figure 11:**
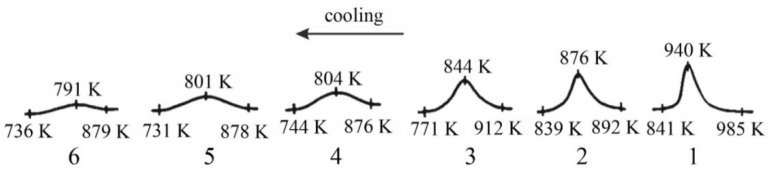
Thermograms recorded for samples 1-6 during the cooling process, showing the temperature of the polymorphic transition from α‘_L_-C_2_S to β-C_2_S.

**Figure 12:**
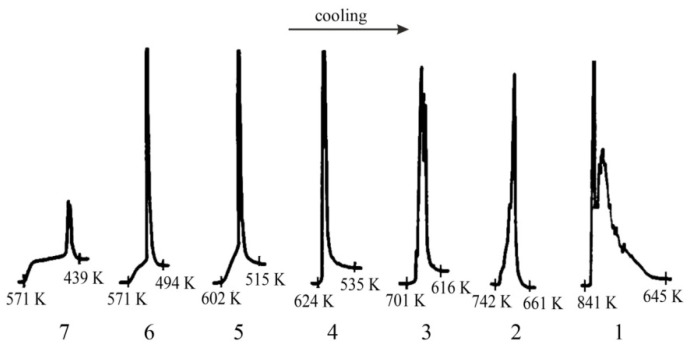
Thermograms recorded for samples 1-7 during the cooling process, showing the temperature of the polymorphic transition from β-C_2_S to γ-C_2_S.

Detailed analyses of the exothermic effects observed for polymorphic transitions from α’_L_-C_2_S to β-C_2_S and from β-C_2_S to γ-C_2_S (self-disintegration) are presented in Figure 11 and Figure 12, respectively. Table 4 lists the heat evolution rates and the mean times required for polymorphic transition to occur, while Table 5 contains data on the amount of heat evolved during polymorphic transitions; both sets of data are shown for all investigated samples.

The multivariate analysis of the amount of heat evolved (calorific value) and the time of polymorphic transition in C_2_S made it possible to quantify the mean rates of the phase transition from α’_L_-C_2_S to β-C_2_S and the transition from β-C_2_S to γ-C_2_S for different samples. The obtained results are presented in Figure 11 and Figure 12. The data show that increased C_12_A_7_ content is associated with lower temperatures at which both the transition from α’_L_-C_2_S to β-C_2_S and the one from β-C_2_S to γ-C_2_S occur.

The DTA results obtained for samples 1–3 indicated that the influence of the C_12_A_7_ phase in the amount in which it was present in these samples on the exothermic effect related to the transition from α’_L_-C_2_S to β-C_2_S was negligible (Figure 11). However, when the C_12_A_7_ content was higher—as for samples 4–6 (taking into account the effect of dilution in the case of sample 6)—the exothermic effect clearly diminished. This thermal effect was observed at increasingly low temperatures. In the case of samples 1–3, the temperature at which the transition from α’_L_-C_2_S to β-C_2_S occurred was 373 K lower than the corresponding temperature for sample 1. The described effect did not occur for samples 7 and 8, as for these samples this type of transition was not observed at all.

The exothermic effect of the transition from β-C_2_S to γ-C_2_S is shown in Figure 12; the close correlation between the course of the disintegration and the magnitude of the exothermic effect as well as the change in the temperature at which it occurs is evident. For increased amounts of the aluminate phase the effect associated with the start of this transition diminishes, and the temperature at which the transition starts decreases by 270 K. For sample 7, the end of the transition occurs at a temperature that is 206 K lower than that for sample 1.

The thermograms shown in Figure 5, Figure 6, Figure 7 and Figure 8 confirm the fact that polymorphic transitions diminish during the cooling of the investigated sinters with high C_12_A_7_ content over the range of 1723–673 K. This dependence is also evident from the results of high-temperature calorimetry (Table 4 and Table 5). The mean heat evolution rate during the transition from α’_L_-C_2_S to β-C_2_S indicates that increasing the C_12_A_7_ content causes this process to decelerate.

The opposite effect is observed in the case of the transition from β-C_2_S to γ-C_2_S, since an increased rate of polymorphic transition connected with self-disintegration is observed for sample 7, for which a four-fold increase in the self-disintegration rate is observed compared to sample 1.

### 3.5. Effect of Cooling Rate on the Transition from β-C_2_S to γ-C_2_S

The influence of the rate at which samples had been cooled on the self-disintegration process was examined via differential thermal analysis over the range from 1723 to 293 K. The cooling rates that were applied were 2.5, 5, 10 and 20 K·min^−1^. Table 6 lists the temperatures at which the transition from β-C_2_S to γ-C_2_S started for selected samples depending on the cooling rate

Samples 1, 4, 6, 7 and 8 were used to determine the influence of the cooling rate on the self-disintegration process. The highest cooling rate of 20 K·min^−1^ was tested first, and each subsequent test involved a lower cooling rate (Table 6).

At the cooling rate of 20 K·min^−1^ self-disintegration started at 822 K for sample 1, whereas for sample 4 the corresponding temperature was lower by as much as 306 K. No self-disintegration was observed for sample 6. Consequently, there was no rationale for continuing the test involving this cooling rate for samples 7 and 8, which contained higher amounts of the silicate phase. At the rate of 10 K·min^−1^, a similar tendency was observed; however, for sample 4, the temperature of the start of self-disintegration was much higher than when the higher cooling rate had been applied, which was not the case for sample 1. For the cooling rate of 5 K·min^−1^ the temperature of the start of self-disintegration decreases with increasing content of the liquid aluminate phase—which was also observed for the two higher cooling rates—but in this case this tendency is also observed for samples 6 and 7. The self-disintegration temperatures recorded for these two samples show that at this point the amount of aluminate phase ceases to affect this parameter. It was decided that further tests involving the lowest cooling rate were unnecessary for samples 1, 4, 6 and 7, as the previous tests had yielded sufficient information as to the tendencies. The only sample that underwent a test at the cooling rate of 2.5 K·min^−1^ was sample 8, and this was done solely because this sample had not undergone self-disintegration at 5 K·min^−1^. However, this test likewise did not result in the self-disintegration of this sample.

The results lead to the conclusion that the amount of aluminate phase does indeed affect the temperature at which the transition from β-C_2_S to γ-C_2_S occurs at a given cooling rate. From a practical standpoint the optimal cooling rate for materials with a C_12_A_7_ content of up to 60 wt% is 5 K·min^−1^.

### 3.6. Grain Size Distribution of Self-Disintegrated Particulates

The results of grain size analysis presented in Table 7 demonstrate that the C_2_S/C_12_A_7_ ratio in sinters has a pronounced effect on the granulometric composition of self-disintegrated particulates. Fine grains represent the largest fraction of the grain population—over 70%—in the case of particulates in which the C_12_A_7_ phase constitutes from 30% to 50% of the total weight. C_12_A_7_ content higher than 50% leads to an increased contribution of thicker grains with a size of over 30 μm.

From the point of view of practical application, the use of materials with 10 or 30 wt% of the liquid aluminate phase makes it easier to obtain fine grains in the 0–30 μm size range and the highest fraction, while at the same time ensuring that undesirable non-disintegrated grains with a size of over 60 μm constitute no more than 0.2% of the total grains.

## 4. Conclusions

The study presents an original attempt to optimize the C_2_S/C_12_A_7_ phase ratio in such a way as to enhance the self-disintegration of C_2_S, which would help reduce the environmental impact and increase sustainability of various industrial and technological processes associated with special cement production. The following conclusions can be made based on the results of the present study:

- Higher aluminate phase (C_12_A_7_) content in C_2_S material leads to a decrease in the temperature at which the polymorphic transitions from α’_L_-C_2_S to β-C_2_S and from β-C_2_S to γ-C_2_S are observed.
- In C_2_S sintered samples with more than 80 wt% of C_12_A_7_, the two investigated phase transitions do not occur in the temperature range of 1600–370 K.
- As the C_12_A_7_ content increases, the amount of heat evolved during the polymorphic transitions from α’_L_-C_2_S to β-C_2_S decreases.
- The time required to fully synthesize C_2_S samples that contain 30 wt% of theC_12_A_7_ phase at 1723 K is below 15 min.
- From a technological standpoint, the optimal cooling rate for C_2_S materials with a C_12_A_7_ content of up to 60 wt% is 5 K·min^−1^.
- The amount of the C_12_A_7_ phase in C_2_S samples has a significant effect on their grain composition. In self-disintegrating samples for which C_12_A_7_ content ranges from 30 to 50 wt% grains with a size of 30 µm or less constitute 70% of the total mass. In the case of C_2_S samples with a C_12_A_7_ content of 50–80 wt%, the mass fraction of undesirable grains with a size of over 60 µm increases.
- The optimal mass ratio of C_2_S/C_12_A_7_ is 70/30, as it ensures both efficient self-disintegration and desirable grain size distribution.
- Self-disintegrating C_2_S with different aluminate phase (C_12_A_7_) content is a promising additive to ordinary Portland calcium aluminate cements, as it may allow the resistance of these cements to chloride- and sulphide-induced as well as biological corrosion to be improved.

## Figures and Tables

**Table 1:** Composition of batches for sinter preparation.

Sample No.	C_2_S [wt%]	C_12_A_7_ [wt%]
1	100	-
2	90	10
3	80	20
4	70	30
5	60	40
6	50	50
7	40	60
8	20	80
9	-	100

**Table 2:** Chemical composition of samples before and after thermal treatment.

Sample No.	Composition before Treatment [wt%]			Composition after Treatment [wt%]		
	CaO	SiO_2_	Al_2_O_3_	CaO	SiO_2_	Al_2_O_3_
1	65.11	34.89	-	65.06	34.94	-
2	63.45	31.39	5.16	63.48	31.09	5.43
3	61.79	27.91	10.30	61.78	27.65	10.57
4	60.13	24.42	15.45	60.19	24.44	15.37
5	58.46	20.93	20.61	58.65	20.43	20.92
6	56.80	17.44	25.76	56.75	17.44	25.81
7	55.14	13.95	30.91	55.24	13.91	30.85
8	51.81	6.98	41.21	51.80	6.94	41.26
9	48.53	-	51.47	48.77	-	51.23

**Table 3:** Free CaO content in selected samples depending on synthesis time.

Sample No.	C_2_S/C_12_A_7_Ratio	Synthesis Time [min]				
		5	15	30	45	60
		Free CaO Content [wt%]				
1	100/-	4.80	4.8	4.40	4.50	4.30
2	90/10	0.80	0.80	0.50	0.30	0.30
4	70/30	0.40	0.10	0.25	0.20	0.20
6	50/50	0.30	0.20	0.15	0.15	0.10
7	40/60	0.30	0.10	0.10	0.10	0.10
8	20/80	0.20	0.10	0.10	0.10	0.10

**Table 4:** Rate of heat evolution and mean time required for the samples to undergo a complete polymorphic transition (N/A—not applicable, polymorphic transition did not occur).

Sample No.	C_2_S/C_12_A_7_Ratio	α‘_L_-C_2_S→β-C_2_S			β-C_2_S→γ-C_2_S		
		Temperature Interval of Transition [K]	Transition Time [s]	Heat Evolution Rate [cal·mol^−1^·s^−1^]	Temperature Interval of Transition [K]	Transition Time [s]	Heat Evolution Rate [cal·mol^−1^·s^−1^]
1	100/-	406	1600	0.30	445	2100	1.5
2	90/10	391	1400	0.30	348	900	2.0
3	80/20	414	1700	0.25	358	1000	2.0
4	70/30	405	1600	0.25	363	1050	2.2
5	60/40	423	1800	0.20	363	1050	2.2
6	50/50	501	1550	0.15	353	920	2.3
7	40/60	N/A	N/A	N/A	303	370	6.1
8	20/80	N/A	N/A	N/A	N/A	N/A	N/A

**Table 5:** Heat evolved during polymorphic transitions (N/A—not applicable, polymorphic transition did not occur).

Sample No.	C_2_S/C_12_A_7_Ratio	α‘_L_-C_2_S → β-C_2_S		β-C_2_S → γ-C_2_S	
		[cal·g^−1^]	[cal·mol^−1^]	[cal·g^−1^]	[cal·mol^−1^]
1	100/-	3.1	532.3	14.1	2430.4
2	90/10	2.4	381.6	10.2	2286.7
3	80/20	2.4	374.6	11.7	2012.3
4	70/30	2.2	375.6	14.0	2408.7
5	60/40	2.2	388.0	13.3	2283.2
6	50/50	1.2	212.6	12.5	2158.2
7	40/60	N/A	N/A	N/A	2250.5
8	20/80	N/A	N/A	N/A	N/A

**Table 6:** Temperature at which the transition from β-C_2_S to γ-C_2_S started for different cooling rates (N/A—not applicable, polymorphic transition did not occur).

Sample No.	C_2_S/C_12_A_7_Ratio	Cooling Rate [K·min^−1^]			
		2.5	5	10	20
		Starting Temperature of β-C_2_S→γ-C_2_S Transition [K]			
1	100/-	-	841	831	822
4	70/30	-	624	599	516
6	50/50	-	571	N/A	N/A
7	40/60	-	571	-	-
8	20/80	N/A	N/A	-	-

**Table 7:** Particle size distribution depending on the C_2_S/C_12_A_7_ ratio in the investigated samples.

Sample No.	C_2_S/C_12_A_7_Ratio	Grain Size [μm]									
		0–10	10–20	20–30	30–40	40–50	50–60	0–20	0–30	30–60	>60
		Fraction of the Total Population [%]									
1	100/-	12.9	14.0	33.5	24.6	10.3	3.7	26.9	59.9	38.6	1.0
2	90/10	8.3	25.7	21.9	49.9	-	-	34.0	55.9	49.9	0.2
3	70/30	17.5	9.7	44.0	12.4	10.0	6.2	27.2	71.2	28.6	0.2
4	50/50	9.1	18.6	43.5	23.1	2.4	1.2	27.6	61.1	25.7	2.1
5	40/60	14.4	12.0	17.9	24.5	14.6	6.6	26.6	45.5	45.7	10.0
6	20/80	-	-	-	-	-	-	-	-	-	-

## Data Availability

Data available on request.

## Supplementary Materials

The following are available online at https://www.mdpi.com/article/10.3390/ma14216459/s1, Video S1: the complete self-disintegration of calcium orthosilicate.
